# High genetic differentiation of Indo‐Pacific humpback dolphins (*Sousa chinensis*) along the Asian Coast of the Pacific Ocean

**DOI:** 10.1002/ece3.8901

**Published:** 2022-05-07

**Authors:** Yufei Dai, Watchara Sakornwimon, Rachawadee Chantra, Liyuan Zhao, Fuxing Wu, Reyilamu Aierken, Kongkiat Kittiwattanawong, Xianyan Wang

**Affiliations:** ^1^ 118477 Laboratory of Marine Biology and Ecology Third Institute of Oceanography Ministry of Natural Resources Xiamen China; ^2^ Key Laboratory of Marine Ecological Conservation and Restoration Ministry of Natural Resources Xiamen China; ^3^ Fujian Provincial Key Laboratory of Marine Ecological Conservation and Restoration Xiamen China; ^4^ Marine and Coastal Resources Research Center The Central Gulf of Thailand Chumphon Thailand; ^5^ Marine and Coastal Resources Research Center The Upper Gulf of Thailand Samut Sakhon Thailand; ^6^ Phuket Marine Biological Research Center Phuket Thailand

**Keywords:** cetacean, genetic diversity, humpback dolphin, microsatellite genotyping, population differentiation

## Abstract

The Indo‐Pacific humpback dolphin (*Sousa chinensis*) is a vulnerable marine mammal species that inhabits shallow, coastal waters from Southeast China, southward throughout Southeast Asia, and westward around the Bay of Bengal to eastern India. Polymorphic microsatellites are useful for elucidating ecological and population genetics‐related questions. Here, 18 new polymorphic microsatellites were developed from *S*. *chinensis* genomic DNA by Illumina MiSeq sequencing. Population genetic analyses were conducted on 42 *S*. *chinensis* individuals from three geographic locations, including the Xiamen Bay of China, the Western Gulf of Thailand, and Andaman Sea. Our microsatellite data revealed a strong and significant population structure among the three sampling regions (overall *F*_ST_ = 0.371, *p* = .001). Pairwise mutual information index also demonstrated high levels of genetic differentiation between different region pairs (values range from 0.272 to 0.339, *p* < .001). Moreover, Structure analysis inferred three genetic clusters, with the high assignment probabilities of 95.92%, 99.47%, and 99.68%, respectively. Principal coordinate analysis plots of individuals divided entire genotypes into three clusters, indicating high level of genetic differentiation. Our results indicated the strong genetic structure in *S*. *chinensis* populations is a result of geographic distances. Other factors such as environmental variables, anthropogenic interference, and social behavior may also have contributed to population differentiation.

## Graphical abstract

The Indo‐Pacific humpback dolphin (*Sousa chinensis*) is a vulnerable marine mammal species that inhabits shallow, coastal waters. Here, 18 polymorphic loci were used for genetic analysis of *S*. *chinensis* individuals from three geographic locations, including the Xiamen Bay of China, the Western Gulf of Thailand, and Andaman Sea. Our microsatellite data reveal a strong and significant population structure among the three sampling regions (overall *F*_ST_ = 0.371, *p* = .001). Structure analysis and principal coordinate analysis plots of individuals divided entire genotypes into three clusters, indicating high level of genetic differentiation.

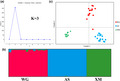

## 1 INTRODUCTION

The Indo‐Pacific humpback dolphin (*Sousa chinensis*) is widely distributed in shallow, coastal waters from Southeast China, southward throughout Southeast Asia, and westward around the Bay of Bengal to eastern India (Jefferson & Curry, 2015; Jefferson & Rosenbaum, 2014; Mendez et al., 2013). Recently, this species has been classified as “vulnerable” on the International Union for Conservation of Nature’s RedList of Threatened Species based on an inferred population size reduction (Jefferson et al., 2017). Primary threats to this vulnerable species are incidental mortality caused by intensive fishing efforts using entangling gear, as well as ongoing habitat loss and degradation due to coastal development. Vessel collisions and environmental contamination may also be significant threats in some areas (Jefferson & Smith, 2016; Jefferson et al., 2017). For long‐lived animals with late maturation and low reproductive rates such as *S*.* chinensis*, these threats often have resulted in priority conservation status being afforded to a number of small and fragmented populations (Brown et al., 2014).

Understanding genetic diversity and population structure is essential for the assessment of conservation status and effective management of a species, especially for inshore dolphins whose isolated populations are highly affected by human activities (Brown et al., 2014; Jefferson et al., 2009; Mace & Lande, 1991). Current information of population parameters is available for only a few sites in China, Malaysia, Thailand, and Bangladesh (Jefferson et al., 2017). Photo‐identification catalogues that would allow the identification of individuals and comparisons between these regions individuals are lacking. Although previous genetic studies revealed strong population structure in different *S*.* chinensis* communities (Amaral et al., 2017; Mendez et al., 2013), the analysis of genetic samples from the Asian coast of the Pacific and the Indian Ocean is still lacking. Recent population‐level analyses based on a single locus of the mitochondrial DNA (mtDNA) control region have detected significant genetic differentiation between most of the geographic populations in both Chinese and Thai waters (Zhao et al., 2021). However, limited conclusions can be drawn when relying on a single mtDNA locus as a marker, because mtDNA is maternally inherited and often has higher mutation rates than nuclear DNA (Mendez et al., 2013). Analyses using additional molecular markers should be conducted.

Microsatellites are widely used and have gradually become an important genetic marker, because highly polymorphic microsatellites are useful for elucidating molecular ecology‐ and population genetics‐related issues such as migration rates, bottlenecks, and kinship (De Barba et al., 2017; Selkoe & Toonen, 2006). These markers are usually short in length (100–300 bp), and they can still be amplified with polymerase chain reaction (PCR), even when using some poor‐quality samples caused by DNA degradation (Taberlet et al., 1999). Microsatellite studies on cetaceans have successfully used genomic DNA extracted from sloughed skin or tissues collected from decomposing stranded animals (Valsecchi & Amos, 1996). However, using low‐quality DNA samples may lead to low amplification success rates and high rates of genotyping errors, such as allelic dropouts and other allele‐like artifacts that are generated by amplification (Bonin et al., 2004; Pompanon et al., 2005).

Traditional methods for microsatellite isolation include construction of an enriched library followed by cloning and Sanger sequencing, which are both expensive and extremely laborious and time‐consuming (Zane et al., 2002). With the development of next‐generation sequencing technologies, isolation of species‐specific microsatellite loci has become more convenient and efficient (Kumar & Kocour, 2017; Vaini et al., 2019). Paired‐end sequencing on the Illumina platform is currently the most commonly used approach for microsatellite isolation (González‐Castellano et al., 2018).

Several genetic and genomic studies have been published on humpback dolphins (Gui et al., 2013; Jia et al., 2019; Ming, Jian, Yu, Yu, et al., 2019; Ming, Jian, Yu, Wang, et al., 2019; Zhang et al., 2020), but only a limited number of microsatellite sequences are reported in *S*.* chinensis*, and some of those are shown to have low polymorphism (Chen & Yang, 2009; Lin et al., 2012). In addition, there are more dinucleotide microsatellites among the existing markers. Using such short tandem repeat motifs may produce a large amount of strand slippage during PCR and increase the likelihood of stutter bands and genotyping errors (Pei et al., 2018; Zalapa et al., 2012). Compared to dinucleotide markers, tetrameric and pentameric markers have lower stutter slippage efficiency and clearer peak discrimination during PCR amplification and genotyping (Gill et al., 2005; Pei et al., 2018).

Therefore, the objectives of this study were to: (1) isolate tetra‐, penta‐, and hexa‐nucleotide microsatellites from *S*.* chinensis* genome sequences using Illumina sequencing; (2) evaluate genetic diversity of *S*.* chinensis* using samples obtained from three different sampling sites along the Asian coast of the Pacific and the Indian Ocean; and (3) infer population structure among the three sampled locations.

## 2 MATERIALS AND METHODS

### 2.1 Ethics permits

All fieldwork was conducted under permits from the Ministry of Agriculture and Rural Affairs of China, and with approval from the Department of Marine and Coastal Resources of Thailand. The relevant CITES Permits (No. 2018CN/IC000475/CH) and HS Code (4103909010) were obtained for import of samples. All samples were collected from dead, stranded individuals.

### 2.2 Sample collection and DNA extraction

**FIGURE 1:**
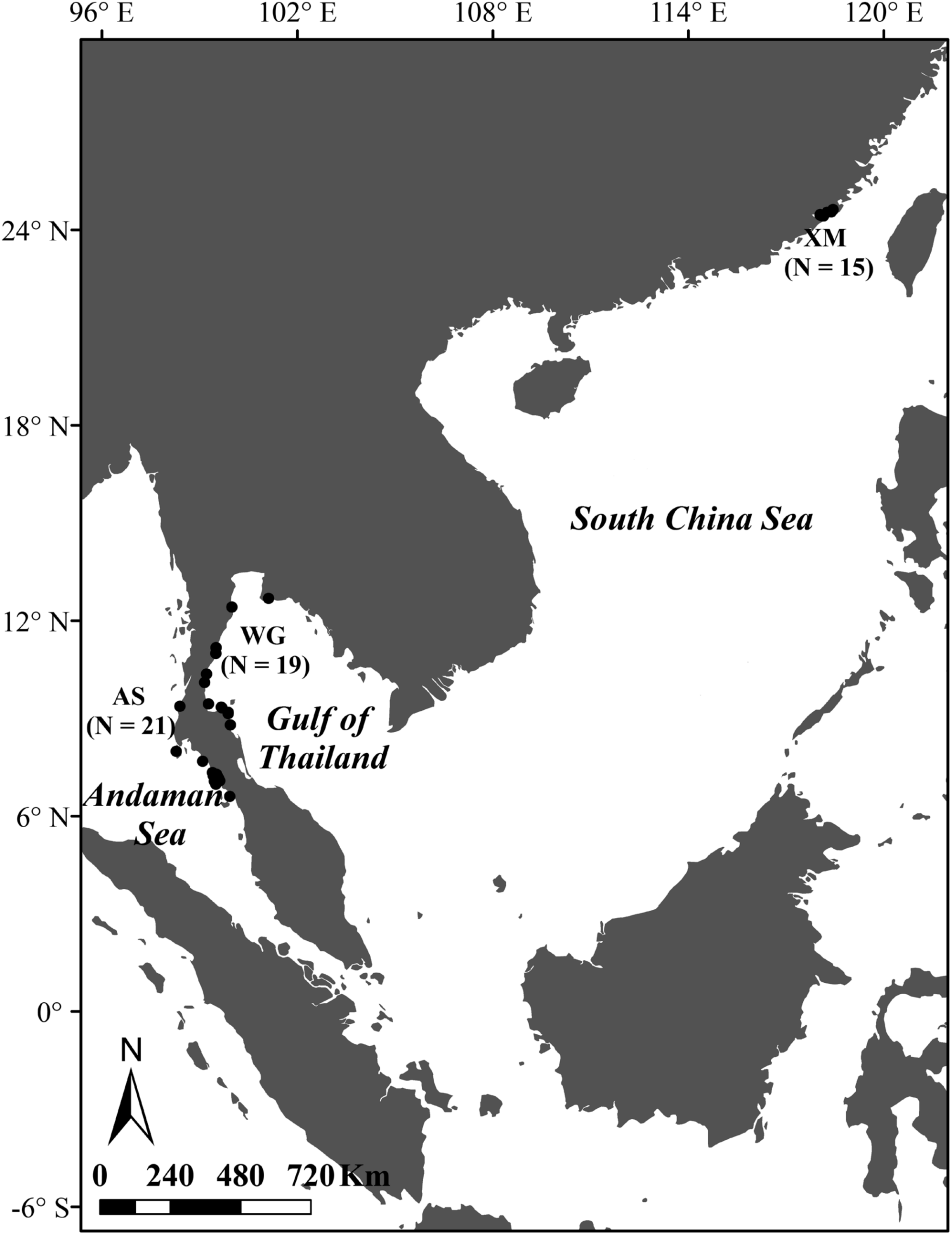
Locations of sample collection in the different geographical regions analyzed in this study. The three sampling sites are including the Xiamen Bay of China (XM, *N* = 15), the western Gulf of Thailand (WG, *N* = 19), and the Andaman Sea site (AS, *N* = 21)

Muscle or skin tissues of 55 dead stranded *S*.* chinensis* were collected from 2010 to 2018, and included 15 individuals obtained along the coast of Xiamen in China (XM), 19 individuals collected from the Western Gulf of Thailand (WG), and 21 individuals collected along the Andaman Sea coast (AS) (Figure 1). Geographic coordinates for most individuals from the three sampling regions were recorded at the time of collection (Appendix S1). Genomic DNA from minced tissue samples were extracted using DNeasy blood and tissue extraction kits (QIAGEN) according to the manufacturer's protocol.

### 2.3 Microsatellite selection and multiplex PCR design

Purified genomic DNA was quantified by TBS‐380 fluorometer (Turner BioSystems Inc.). High‐quality DNA (OD 260/280 = 1.8–2.0, >1 μg) of a single individual collected at XM was used to generate an enriched library and sequenced on the Illumina MiSeq platform by Majorbio Bio‐Pharm Technology Co., Ltd. The detailed procedures were as follows. First, the DNA sample was sheared into 400–500‐bp fragments using a Covaris M220 Focused Acoustic Shearer following the manufacturer’s protocol. Then, an Illumina sequencing library was prepared from the sheared fragments using the NEXTflex™ Rapid DNA‐Seq Kit. Briefly, 5′ ends were first end‐repaired and phosphorylated. Next, the 3′ ends were A‐tailed and ligated to the sequencing adapters. The third step was to enrich the adapter‐ligated products using PCR. Finally, the prepared library was used for paired‐end Illumina sequencing (2 × 150 bp) on an Illumina HiSeq X Ten machine. After filtering low quality and duplicated sequences and removing adapter‐related reads, a total of 2,174,959 clean reads were assembled using SOAPdenovo version 2.04 software (Luo et al., 2012). At last, 1,398,738 contigs including 95,161 large contigs (>1000 bp) were obtained, with an average GC content of 55.49% and contig N50 of 1513 bp (Table 1).

**TABLE 1:** Summary of dataset assemblies for *S*.* chinensis* through Illumina sequencing

Assembly statistics	Data statistics
Total number of contigs	1,398,738
Total bases of contigs	724,659,648
Total number of large contigs (>1000 bp)	95,161
Total bases of large contigs	148,023,926
Contig N50 (bp)	1513
G+C content (percentage)	55.491%

**FIGURE 2:**
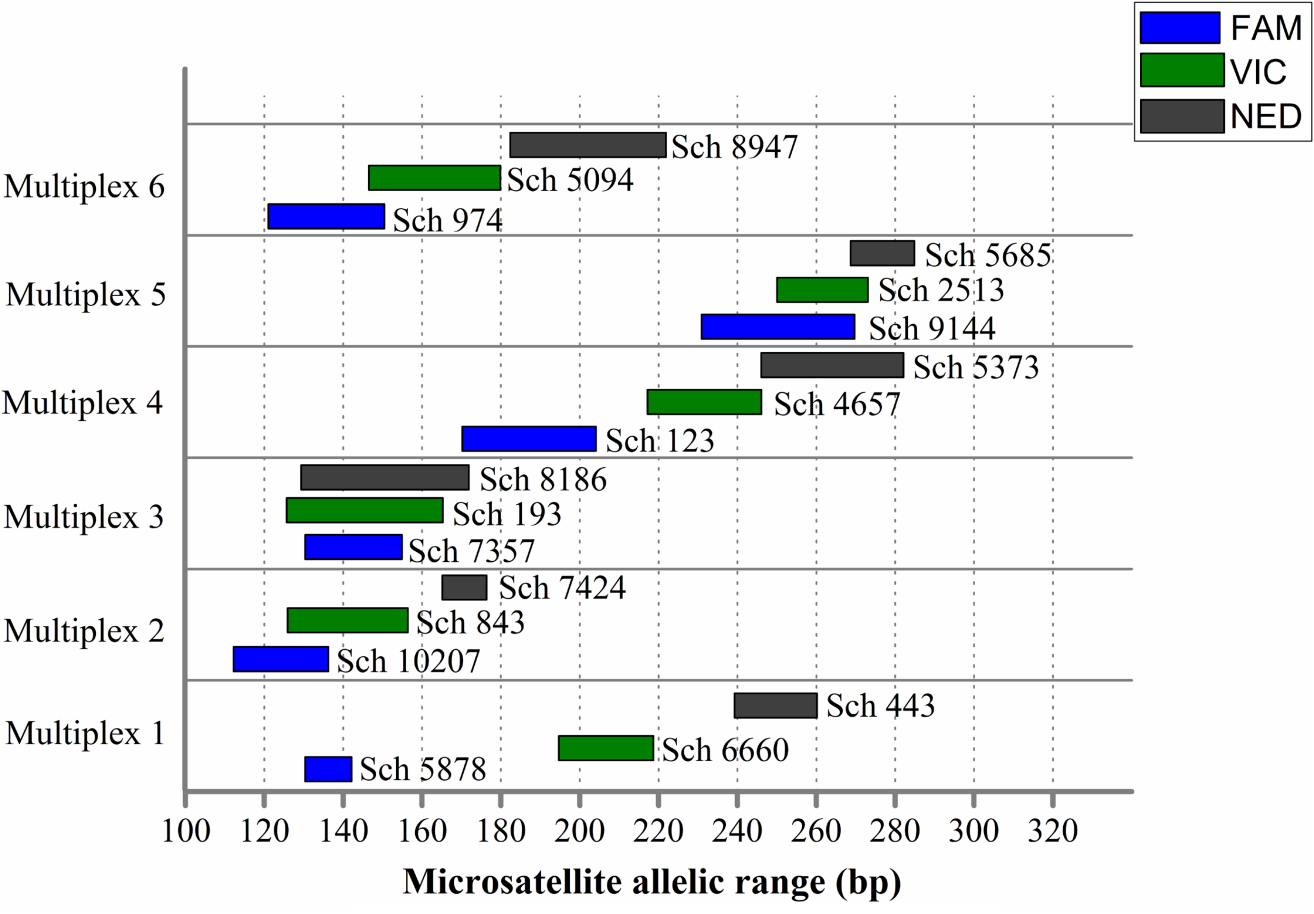
The 18 polymorphic microsatellite markers allocated in six multiplex PCR panels. Each locus is shown by allelic range, and different colors represent different fluorescent dyes (FAM: blue, VIC: green, NED: black)

The assembled data were searched for tetra‐, penta‐, and hexa‐nucleotide microsatellite motifs using MSATCOMMANDER version 0.8.2 (Faircloth, 2008). The searching parameters were a minimum of 10 repeats for tetra‐nucleotide motifs, and six repeats for the other two repeat classes. Only ‘perfect‐type’ microsatellite sequences (pure repeats) with a flanking region of at least 30 bp on each side were selected. PCR primers for 162 available microsatellites were designed using Primer3 (Rozen & Skaletsky, 2000). After primers design and PCR genotyping, a total of 18 polymorphic microsatellite loci (Table 2) with ‘perfect‐type’ and long tandem repeat motifs were allocated into 6 multiplex PCR panels using software MPprimer (Shen et al., 2010), based on annealing temperature, complementarity of primer pairs, and allele size range (Figure 2).

**TABLE 2:** Multiplex design information for all the tested 18 microsatellite loci in *S*.* chinensis*. Primer concentration was 0.2 μM

Multiplex panels	Locus	Repeat motif	Primer sequences (5′−3′)	Primer dosage (µl)	Annealing temperature (℃)	GenBank accession number	Fluorescent label
Multiplex 1	Sch5878	(CAAC)_12_	F: TCTCCAGTGTTTGGGCTCTT	0.20	61	MK766860	6‐FAM
			R: ACATTTTGGAAGGCAAGCTG	0.20			
	Sch6660	(AAGG)_13_	F: CTGAGTGGTCCTCAAGGGAG	0.18		MK766861	VIC
			R: TCTGCTGACATGCCTCACTC	0.18			
	Sch443	(CCAT)_12_	F: GGACTACAAGAAGCTGGGCA	0.18		MK766850	NED
			R: CTGGTGCGTGTAGCTGTTGT	0.18			
Multiplex 2	Sch10207	(CATC)_12_	F: CCCTCTCTTGCTCTCTCCCT	0.15	62	MK766870	6‐FAM
			R: TGTCTATTGTACAGCAGGATGGA	0.15			
	Sch843	(AAAT)_11_	F: GAGAAACATTTTGTCTAAGTGCTCTG	0.15		MK766851	VIC
			R: GAACGCAGATCCTAACGTCTAATTAG	0.15			
	Sch7424	(ATGG)_13_	F: GGAAGGGTGGATGGTTAGGT	0.15		MK766864	NED
			R: ATGTTCCCTGAGGATTGTGC	0.15			
Multiplex 3	Sch7357	(ATGG)_11_	F: CAGTGCCTCGAACAGAGATTG	0.15	61	MK766863	6‐FAM
			R: AAGTATTCCCACACCCATCCA	0.15			
	Sch193	(AGAGA)_12_	F: GTATGGAAGGAAGGGAGGGA	0.20		MK766846	VIC
			R: CAAACTAAGGAAGCAAATGCAG	0.20			
	Sch8186	(CCAT)_11_	F: CCCACAGAAGTCAAGCATCA	0.15		MK766865	NED
			R: CTGGAATCTGGGGTGAGAAAT	0.15			
Multiplex 4	Sch123	(CCTAAC)_7_	F: GGAAGCCAGGTACCACTGTTG	0.15	63	MK766845	6‐FAM
			R: TGAGGACAGCACAGACCAGAG	0.15			
	Sch4657	(TTCC)_11_	F: TGTCCATGCAAGCGTAAATC	0.15		MK766855	VIC
			R: AGTGTTGGCATTTCTCCAGC	0.15			
	Sch5373	(GATG)_11_	F: GGCTCCAGAGCTTGTGATCT	0.15		MK766858	NED
			R: GGAAGTCCATCTCCCTCTCC	0.15			
Multiplex 5	Sch9144	(ATCT)_14_	F: TAGAGCCTGCATGAGTGTGG	0.15	59	MK766868	6‐FAM
			R: CAACAGAGTCAGCGTGCCT	0.15			
	Sch2513	(CATC)_13_	F: GGGTTTACACCTGTCGCTGT	0.15		MK766854	VIC
			R: TCAACACATCATTGCCGAAT	0.15			
	Sch5685	(AGGA)_12_	F: CATTCTTCCAGATGTACGTCCA	0.25		MK766859	NED
			R: CCTCGGGTAAGTCCCTCTTC	0.25			
Multiplex 6	Sch974	(GTTTT)_13_	F: GCTGAGGATATCAGGGTGGA	0.15	59	MK766849	6‐FAM
			R: CAGGGAAGTCCCAGAAATCA	0.15			
	Sch5094	(TCTA)_11_	F: CTGGGGTTTCTAGCTTGCAG	0.15		MK766857	VIC
			R: ATTCTCCAGAGGAACCAGCA	0.15			
	Sch8947	(CTAT)_12_	F: GGGAAAGATGCCAATCTGAA	0.25		MK766867	NED
			R: CGTACCGCAACAAAGAGTGA	0.25			

### 2.4 Microsatellite genotyping

The 5′ end of each forward primer was labeled with a fluorescent dye (6‐FAM, VIC or NED). The total PCR volume (20 μl) consisted of approximately 50 ng of genomic DNA, 1×Multiplex PCR Kit (Takara), 0.2 μM of each primer (forward and reverse), and ddH_2_O added to make up the final volume (Table 2). PCR conditions involved an initial denaturation step at 94°C for 3 min, followed by 32 cycles of 94°C for 30 s, the specific annealing temperature (Table 2) for 90 s, extension at 72°C for 60 s, and a final extension for 30 min at 60°C. Fragment analysis was performed on the PCR products on an ABI 3730XL automated DNA sequencer (Applied Biosystems), using GeneScan LIZ 500 as the internal size standard. Allele sizes were automatically scored with GeneMapper version 4.1 (Applied Biosystems) and manually checked. We used the same individual as a positive control for genotyping each locus separately in each multiplex PCR panel to ensure consistent amplification of alleles. A negative control without DNA template was also used in each PCR batch to detect possible contamination during PCR amplification. Moreover, genotypes from six individuals (>10%) were randomly retested to estimate genotyping error rates for across all 18 loci. Samples of each of the six samples were re‐amplified and resequenced.

### 2.5 Statistical analysis

GenAIEx version 6.501 (Peakall & Smouse, 2006, 2012) was used to estimate the genetic parameters for each sampling location, including the number of alleles per locus (Na), effective number of alleles (eNa), observed heterozygosity (Ho), expected heterozygosity (He), unbiased expected heterozygosity (uHe), and Shannon′s information index (I). GENEPOP version 4.0.7 (Rousset, 2008) was used to test departure from Hardy–Weinberg equilibrium (HWE) and linkage disequilibrium (LD) among all pairs of loci. The *p*‐value‐based multiple testing of Bonferroni sequential correction was performed with Myriads version 1.1 (Carvajal‐Rodriguez, 2018). Micro‐Checker version 2.2.3 (Van Oosterhout et al., 2004) was used to detect occurrences of null alleles, allelic dropouts, or scoring error for each locus, with 95% confidence intervals.

FSTAT version 2.9.3.2 (Goudet, 1995, update in Feb. 2002) was used to assess the estimator of genetic population differentiation, *F*_ST_ (Weir & Cockerham, 1984), among different sampling sites based on 1000 permutations. In addition, the mutual information index (sHua) (Sherwin et al., 2006) between different region pairs was estimated using GenAlEx software.

We inferred population structure, using Structure version 2.3.4 (Pritchard et al., 2000), which estimates the number of genetic clusters (*K*) based on genotyping data generated from the six multiplex PCR panels. The length of the burn‐in period was set to 10^5^ iterations, followed by 10^6^ in the number of Markov Chain Monte Carlo iterations. The LOCPRIOR model was chosen to infer possible weak population structure with the assistance of sample group information. The number of inferred *K* was set between 1 and 10, and 20 independent replicates were run for each *K* value. Subsequently, the Structure Harvester version 0.6.94 (Earl & VonHoldt, 2012) online tool was used to calculate the Delta *K* value and determine the best number of *K* clusters (Evanno et al., 2005). CLUMPP version 1.1.2 (Jakobsson & Rosenberg, 2007) was used to summarize the optimal alignment of the 20 replicates for the same *K* value. The final results were displayed graphically with Distruct version 1.1 (Rosenberg, 2004).

Moreover, a principal coordinate analysis (PCoA) was performed in GenAIEx based on the standardized covariance of the individual‐by‐individual genetic distance matrix. Mantel analysis (Diniz‐Filho et al., 2013) was also used in GenAIEx to test isolation by distance (IBD) by testing for correlation between matrices of individual‐by‐individual genetic distances and geographic distances measured as the distance between two individuals calculated from sampling location coordinates. We conducted a second IBD analysis excluding the genetic and geographical data for XM samples because of the large geographic distances between XM and other regions. Both tests were run with 999 random permutations in GenAIEx.

## 3 RESULTS

### 3.1 Available microsatellite data for analysis

**FIGURE 3:**
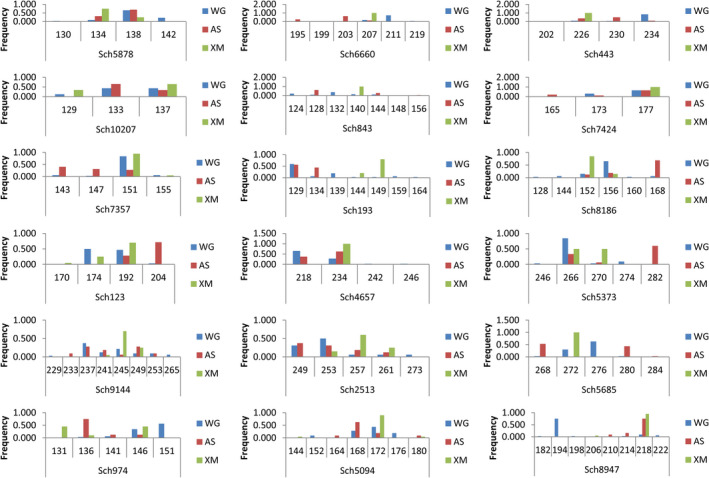
Allele Frequencies with Graphs by each sampling location and each locus for microsatellite data. Allele frequencies displayed for 18 polymorphic microsatellites, and different colors represent different sampling regions (WG: blue, AS: red, XM: green)

For genetic analysis, only DNA samples for which at least 16 out of the 18 loci could be genotyped were included. Therefore, 13 samples of individuals (five from XM, three from WG and five from AS) were discarded because of poor amplification success. For the remaining 42 individuals, no contamination was detected during multiple PCRs, and no genotyping errors were observed when randomly retesting the six individuals. Finally, genotypes of 18 microsatellites for 42 individuals were used in this genetic study, including 10 individuals from XM, 16 from WG, and 16 from AS. There was no evidence of allelic dropouts or scoring errors due to stuttering for any of the 18 loci in all the three sampling locations. Allele frequency distributions of different sampling regions based on 18 polymorphic loci are graphically represented in Figure 3.

### 3.2 Genetic diversity

**FIGURE 4:**
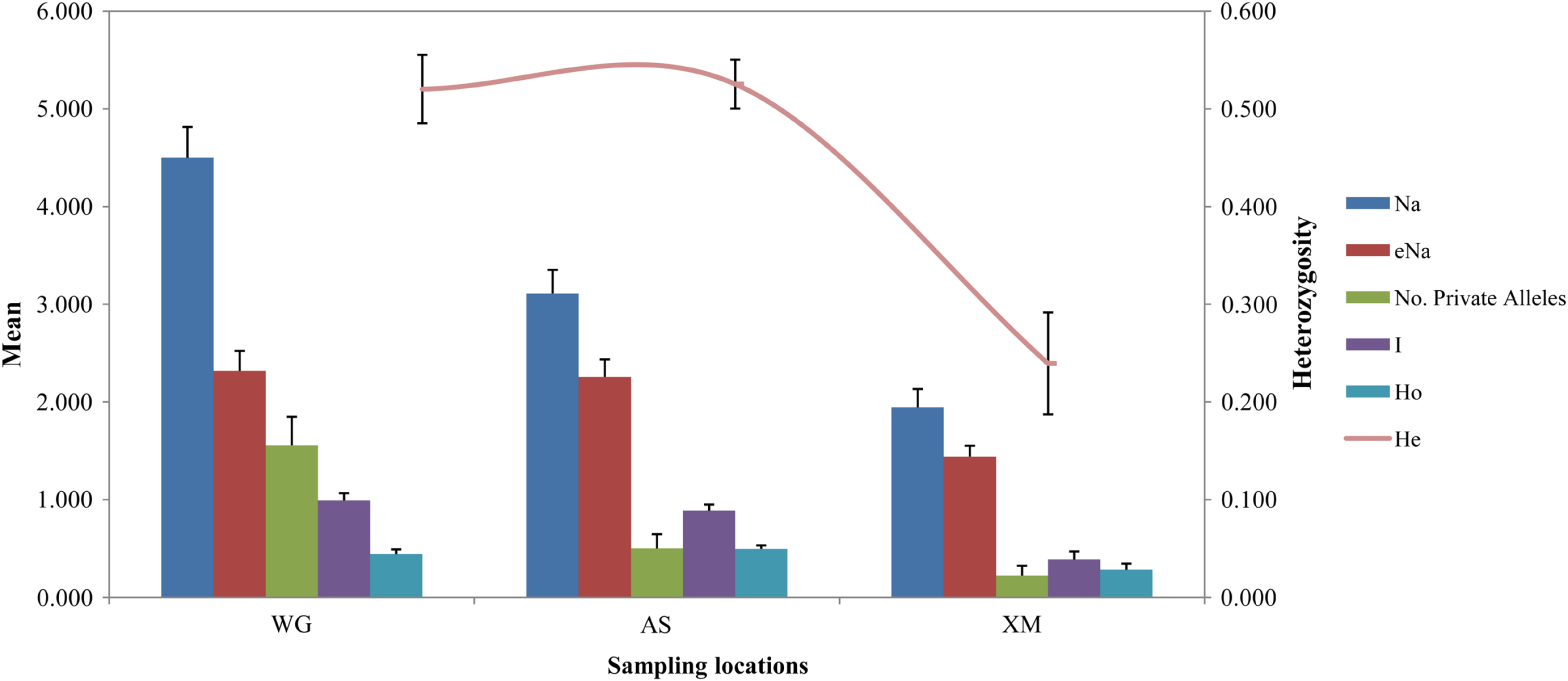
Allelic patterns across populations in the geographic distribution of *S*.* chinensis*. Na is the number of different alleles, eNa is the effective number of alleles, I is the Shannon's information index, Ho is the observed heterozygosity, and He represented by the curve is the expected heterozygosity. The bars represent Standard Error (SE) values

Genetic diversity estimates of Na, eNa, I, Ho, He, and uHe values for each locus and each region are summarized in Table 3. The average estimates of Na, eNa, and I were highest in WG, with values of 4.500 ± 0.316, 2.319 ± 0.204, and 0.991 ± 0.076, respectively. The highest mean Ho and He were in AS, with values 0.497 ± 0.035 and 0.525 ± 0.025, respectively (Figure 4). ANOVA results revealed Na values differed significantly from each other among sampled regions (ANOVA: *F*_2, 51_ = 25.372, *p* < .001). There were also significant differences among eNa (*F*_2, 51_ = 8.430, *p* = .001), I (*F*_2, 51_ = 19.031, *p* < .001) and Ho (*F*_2, 51_ = 4.850, *p* = .012) values. Additionally, significant differences were found among He (*F*_2, 51_ = 17.556, *p* < .001) and uHe (F_2, 51_ = 16.600, *p* < .001) from any of the three geographic regions. The results revealed that the levels of genetic diversity detected for *S*.* chinensis* in XM were the lowest.

**TABLE 3:** Genetic diversity parameters in the three geographic regions. *N* is the sample size, Na is the number of alleles, eNa is the number of effective alleles, I is the Shannon′s information index, Ho is the observed heterozygosity, He is the expected heterozygosity, uHe is the unbiased expected heterozygosity, P_HWE_ is the *p* value of Hardy‐Weinberg equilibrium test, * indicates significant departure from Hardy–Weinberg equilibrium after sequential Bonferroni correction (*p* < .003), ND represents not done

Pop	Locus	*N*	Na	eNa	I	Ho	He	uHe	P_HWE_
WG	Sch5878	16	4.000	2.048	0.939	0.438	0.512	0.528	0.096
	Sch6660	16	4.000	1.796	0.833	0.188	0.443	0.458	0.001*
	Sch443	16	3.000	1.380	0.539	0.250	0.275	0.284	0.246
	Sch10207	16	3.000	2.510	0.983	0.438	0.602	0.621	0.299
	Sch843	16	6.000	4.197	1.580	0.813	0.762	0.786	0.457
	Sch7424	16	3.000	1.889	0.748	0.375	0.471	0.486	0.414
	Sch7357	16	4.000	1.388	0.598	0.063	0.279	0.288	0.000*
	Sch193	16	7.000	2.510	1.295	0.500	0.602	0.621	0.022
	Sch8186	16	6.000	2.151	1.130	0.500	0.535	0.552	0.005
	Sch123	16	3.000	2.124	0.810	0.563	0.529	0.546	0.747
	Sch4657	16	4.000	1.954	0.850	0.500	0.488	0.504	0.000*
	Sch5373	16	4.000	1.384	0.582	0.313	0.277	0.286	0.997
	Sch9144	16	7.000	4.414	1.686	0.813	0.773	0.798	0.144
	Sch2513	16	5.000	2.783	1.230	0.500	0.641	0.661	0.021
	Sch5685	15	4.000	2.027	0.877	0.333	0.507	0.524	0.603
	Sch974	16	4.000	2.276	0.972	0.625	0.561	0.579	0.008
	Sch5094	16	4.000	3.180	1.254	0.625	0.686	0.708	0.267
	Sch8947	16	6.000	1.730	0.936	0.125	0.422	0.435	0.000*
	Mean	15.944	4.500	2.319	0.991	0.442	0.520	0.537	
AS	Sch5878	16	2.000	1.753	0.621	0.375	0.430	0.444	0.611
	Sch6660	16	3.000	2.133	0.900	0.563	0.531	0.548	0.948
	Sch443	16	4.000	2.498	1.045	0.625	0.600	0.619	0.289
	Sch10207	16	2.000	1.822	0.643	0.563	0.451	0.466	0.324
	Sch843	16	4.000	2.107	0.932	0.500	0.525	0.542	0.448
	Sch7424	16	3.000	2.024	0.869	0.625	0.506	0.522	0.652
	Sch7357	16	3.000	2.926	1.086	0.625	0.658	0.679	0.548
	Sch193	16	2.000	1.969	0.685	0.375	0.492	0.508	0.341
	Sch8186	16	3.000	1.910	0.831	0.375	0.477	0.492	0.311
	Sch123	16	2.000	1.679	0.594	0.563	0.404	0.417	0.118
	Sch4657	16	2.000	1.882	0.662	0.500	0.469	0.484	0.790
	Sch5373	15	3.000	2.103	0.853	0.133	0.524	0.543	0.002*
	Sch9144	16	6.000	4.655	1.645	0.813	0.785	0.810	0.063
	Sch2513	16	4.000	3.459	1.305	0.438	0.711	0.734	0.007
	Sch5685	16	3.000	2.107	0.806	0.500	0.525	0.542	0.718
	Sch974	16	3.000	1.684	0.736	0.313	0.406	0.419	0.217
	Sch5094	16	4.000	2.256	1.051	0.563	0.557	0.575	0.851
	Sch8947	16	3.000	1.679	0.728	0.500	0.404	0.417	0.620
	Mean	15.944	3.111	2.258	0.888	0.497	0.525	0.542	
XM	Sch5878	10	2.000	1.600	0.562	0.500	0.375	0.395	0.292
	Sch6660	10	1.000	1.000	0.000	0.000	0.000	0.000	ND
	Sch443	10	1.000	1.000	0.000	0.000	0.000	0.000	ND
	Sch10207	10	2.000	1.835	0.647	0.700	0.455	0.479	0.089
	Sch843	10	1.000	1.000	0.000	0.000	0.000	0.000	ND
	Sch7424	10	1.000	1.000	0.000	0.000	0.000	0.000	ND
	Sch7357	10	2.000	1.105	0.199	0.100	0.095	0.100	0.868
	Sch193	10	2.000	1.471	0.500	0.400	0.320	0.337	0.429
	Sch8186	10	2.000	1.342	0.423	0.300	0.255	0.268	0.577
	Sch123	10	3.000	1.802	0.746	0.600	0.445	0.468	0.607
	Sch4657	10	1.000	1.000	0.000	0.000	0.000	0.000	ND
	Sch5373	10	2.000	2.000	0.693	0.600	0.500	0.526	0.527
	Sch9144	10	3.000	1.802	0.746	0.400	0.445	0.468	0.873
	Sch2513	10	3.000	2.247	0.938	0.600	0.555	0.584	0.343
	Sch5685	10	1.000	1.000	0.000	0.000	0.000	0.000	ND
	Sch974	10	3.000	2.410	0.949	0.600	0.585	0.616	0.989
	Sch5094	10	3.000	1.227	0.394	0.200	0.185	0.195	0.989
	Sch8947	10	2.000	1.105	0.199	0.100	0.095	0.100	0.868
	Mean	10.000	1.944	1.441	0.389	0.283	0.239	0.252	

### 3.3 Hardy–Weinberg equilibrium and linkage disequilibrium

The results of the HWE tests showed no significant deviations (*p* > .05) in XM. Six loci (Sch443, Sch843, Sch4657, Sch5685, Sch6660, and Sch7424) were monomorphic. All the 18 loci showed polymorphism in WG and AS. One locus (Sch5373) in AS and four loci (Sch4657, Sch6660, Sch7357, and Sch8947) in WG showed significant heterozygosity deficits after sequential Bonferroni correction (Table 3). No significant LD was found in any of the 153 pairs of the 18 tested loci after Bonferroni sequential correction for multiple tests.

### 3.4 Genetic differentiation

Genetic differentiation among different sampled locations was estimated by comparing pairwise *F*_ST_ and sHua values that were calculated based on genetic data from the 18 microsatellites. The estimated *F*_ST_ revealed significant and strong genetic differentiation (overall *F*_ST_ = 0.371, *p* = .001) among the three geographic regions. The pairwise sHua ranged from 0.272 to 0.339, which also demonstrated high levels of genetic differentiation between different region pairs (*p* < .001). Estimates of pairwise *F*_ST_ and sHua values are presented in Table 4. AMOVA results for the degree of variance in *S*.* chinensis* individuals are summarized in Table 5. There was 37.08% genetic variance among the three geographic regions, 6.85% variance among individuals within each region, and 56.07% variance within individuals.

**TABLE 4:** Matrix of pairwise mutual information index (sHua, above diagonal) and *F*_ST_ (below diagonal) estimates among the three geographic regions based on microsatellites

Sampling region	XM	WG	AS
XM	–	0.293***	0.339***
WG	0.422***	–	0.272***
AS	0.445***	0.290***	–

*** *p* < .001.

**TABLE 5:** Analysis of molecular variance of *S*.* chinensis* in the three sampled regions. df is the degrees of freedom, SS is the sums of squares, MS is the mean squares, Est. Var. is the estimated variance within and among populations

Source of variation	df	SS	MS	Est. Var.	%
Among populations	2	148.144	74.072	2.527	37.078
Among individuals with population	39	185.475	4.756	0.467	6.854
Within individuals	42	160.500	3.821	3.821	56.068
Total	83	494.119		6.816	100

**FIGURE 5:**
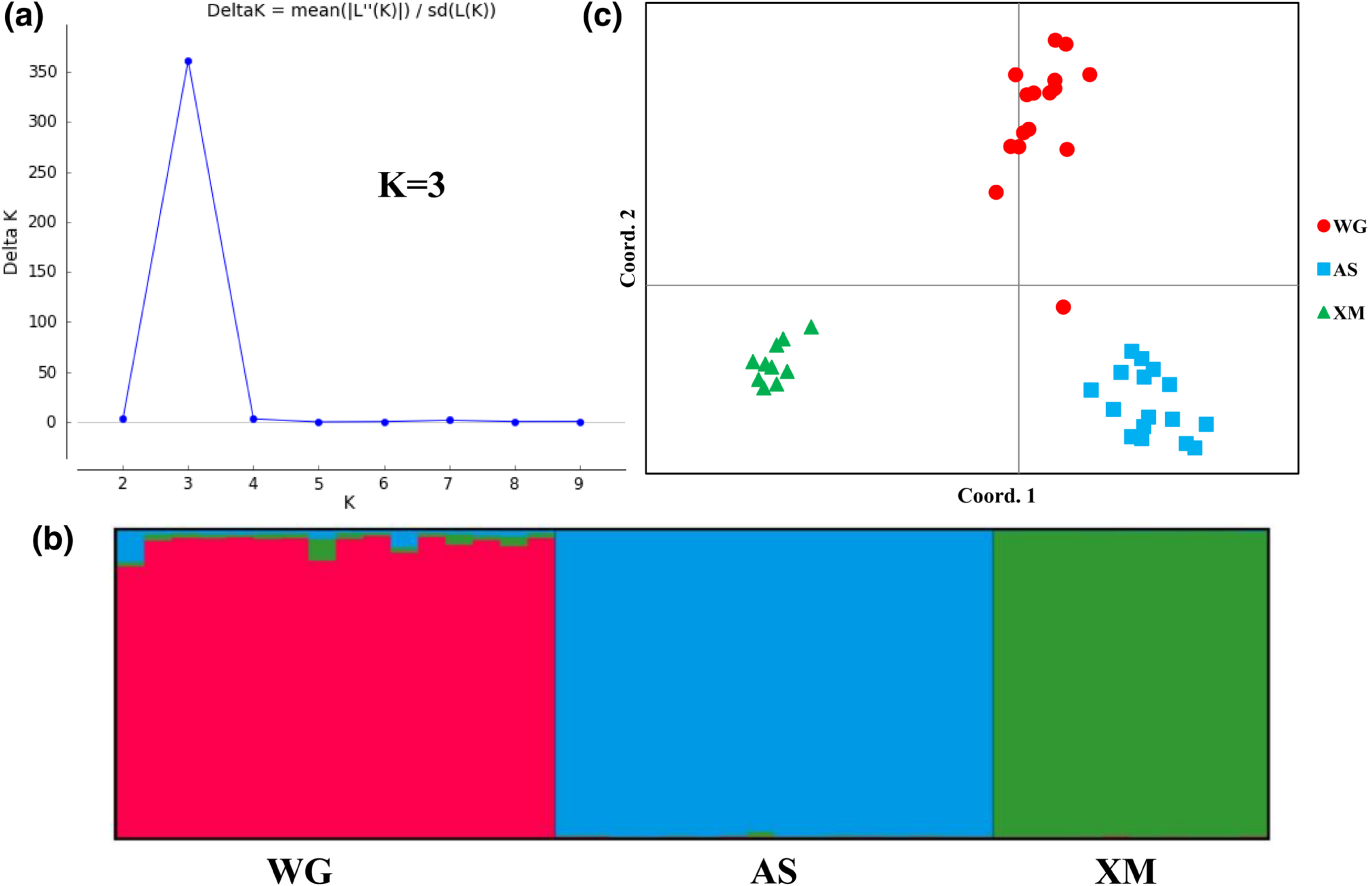
Population structure and Principal Coordinates (PCoA) plot among 42 genotypes of *S*.* chinensis* individuals based on 18 microsatellite markers. (a) Optimal *K* value determined by Structure Harvester online program; (b) Results of clustering (*K* = 3) calculated by Structure program based on the individual *Q*‐matrix. Black vertical lines separate the three sampling locations, and different colors represent the possible genetic clusters; (c) PCoA plot of individuals based on the standardized covariance of genetic distance matrix. Different shapes and colors represent different sampling regions (WG: red circle, AS: blue square, XM: green triangle)

Structure Harvester analysis showed a clear peak for Delta *K* at *K* = 3 (Figure 5a), indicating that there were three clusters based on 42 genotypes of *S*.* chinensis* individuals in the regions that were sampled. The graphical output by Distruct suggested a division into three distinct clusters with no admixture among populations (Figure 5b). For *K* = 3, a strong genetic structure for the three inferred genetic clusters (XM, WG, and AS) was apparent, with the high assignment probabilities of 95.92%, 99.47%, and 99.68%, respectively. No possible partitions within cluster were detected. PCoA plots of individuals based on standardized covariance of the genetic distance matrix also divided all genotypes into three clusters, indicating a high level of genetic differentiation (Figure 5c). Axis coordinates 1 and 2 accounted for 24.93% and 22.10% of the total variance, respectively.

**FIGURE 6:**
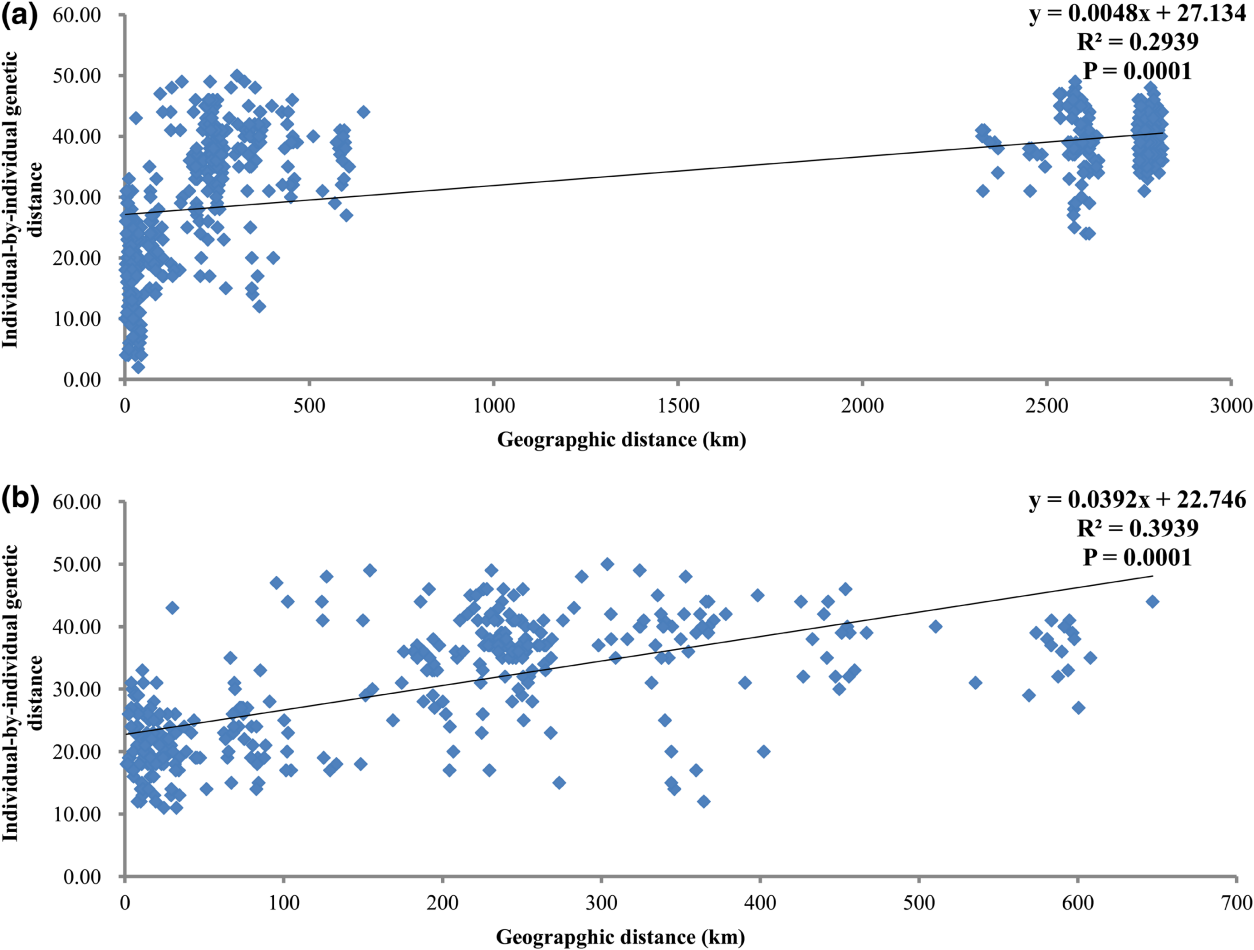
Isolation by distance plots using individual‐by‐individual genetic distances and geographic distances (km) among (a) the three (WG, AS, and XM), and (b) the two (WG and AS) sampling regions. Geographic and microsatellite data of 35 individuals were included in analysis, including 11 individuals from WG, 16 from AS, and 8 from XM

The Mantel tests revealed a positive and significant correlation (*R* ^2^ = .2939, *p* = .0001) between the individual‐by‐individual genetic distances and the geographic distances, indicating a pattern of IBD among the three geographic regions (Figure 6a). The result remained positive when geographic coordinates and microsatellite data for XM individuals were removed (*R* ^2^ = .3939, *p* = .0001; Figure 6b).

## 4 DISCUSSION

In this study, 18 polymorphic microsatellites with tetra‐, penta‐, and hexa‐nucleotide repeats were isolated from the genomic DNA of *S*.* chinensis* and used for genetic analysis of 42 *S*.* chinensis* individuals from three geographic locations. Dimeric and trimeric microsatellite loci were not considered in this work, because stutter bands caused by slipped strand mispairing during PCRs might have occurred at short tandem repeat motifs (Hauge & Litt, 1993; Murray et al., 1993). Compared with dimeric microsatellites, tetrameric and pentameric loci are shown to have lower stutter slippage efficiency and clearer peak discrimination during PCR amplification and genotyping (Pei et al., 2018). Here, we provided 18 novel polymorphic microsatellite markers, which could be useful for future molecular genetics studies on *S*.* chinensis* and other closely related species.

Estimating the levels of genetic diversity in natural populations provides important information for evaluating species viability (An et al., 2014). Our microsatellite data showed the levels of genetic diversity detected for *S*.* chinensis* in XM were significantly lower than those in the other two populations. The low genetic diversity in XM may be related to the small sample size. However, previous studies have revealed low levels of molecular diversity in *S*.* chinensis* in Chinese waters (Chen et al., 2008, 2010; Lin et al., 2010, 2012; Zhang et al., 2020; Zhao et al., 2021). Moreover, mtDNA data of humpback dolphins in China have displayed the lowest genetic diversity, despite having the largest sample size among all sampled regions (Amaral et al., 2017; Mendez et al., 2013). Historical bottlenecks that lead to a reduction in population size may explain such low genetic diversity in humpback dolphin communities in China (Lin et al., 2010). Besides, habitat loss caused by human development has been largely responsible for the decline of *S*.* chinensis* populations in southern China (Lin et al., 2016; Wang et al., 2017). In Xiamen Bay, photo‐identification surveys have recorded only a few *S*.* chinensis* individuals (approximately 60 individuals) across seasons from 2010 to 2015 (Wang et al., 2016; Zeng et al., 2020). By contrast, the minimum population size of *S*.* chinensis* off Donsak in the Western Gulf of Thailand was estimated to be 193 during the survey period from 2011 to 2013 (Jutapruet et al., 2015). The low genetic diversity in *S*.* chinensis* in XM may be associated with random genetic drift in this small population. More attention should be paid to conservation management of *S*.* chinensis* in XM, because genetic deterioration and stochastic events can significantly increase the risk of random extinction in the small populations (Karczmarski et al., 2017).

The results revealed a significant strong genetic differentiation in *S*.* chinensis* among different regions for both *F*_ST_ and sHua estimates. A previous study revealed that *F*_ST_ was more appropriate than other estimators when numbers of loci and sample size were limited (Balloux & Goudet, 2002). Compared with *F*_ST_, the sHua value is known to be a better estimator of genetic differentiation, and can robustly reflect dispersal over a wide range of population sizes and dispersal rates (Manlik et al., 2019; Sherwin et al., 2017). Similarly, our results of the estimated sHua also showed a high degree of genetic differentiation, which indicates a strong population structure among *S*.* chinensis* communities of these three sampled regions. Relatively higher degrees of genetic differentiation with higher pairwise *F*_ST_ or Φ_ST_ values (>0.5) have been reported for *S*.* chinensis* between populations in China and Thailand based on mtDNA sequence data (Amaral et al., 2017; Mendez et al., 2013; Zhao et al., 2021).

The Mantel analysis showed a pattern of IBD among different sampling regions, which indicated that the observed genetic differentiation in *S*.* chinensis* communities is a result of geographic distances. Phylogenetic analysis of the *Sousa* genus based on both mtDNA and nuclear DNA markers demonstrated that the Chinese and Thai haplotypes represented one assemblage, although morphological evidence revealed a clear distinction between the two sampling regions (Mendez et al., 2013). Theoretically, there is still potential genetic exchange or contact between *S*.* chinensis* communities in China and Thailand. However, humpback dolphins are known to prefer shallow waters (less than approximately 25 m in depth) and reside only in coastal (generally within 1–2 km off shore) and estuarine waters (Hung & Jefferson, 2004; Jefferson, 2000; Jefferson et al., 2009). This species shows minimal linear distance movement, with a maximum dispersal distance of 300 km in Chinese waters (Jefferson & Hung, 2004; Wang et al., 2016). Therefore, exchange of *S*.* chinensis* individuals seems to more likely occur between adjacent communities in China. The geographic distance from the northern South China Sea to the Gulf of Thailand is over 3000 km, which is much greater than the *S*.* chinensis* individual dispersal distance. Our microsatellite data also revealed that the genetic structure of *S*.* chinensis* in China and Thailand follows an IBD model, which can explain the strong genetic differentiation among the three sampling locations in this study.

The possible mechanisms that drive the population differentiation and even result in the species boundaries of humpback dolphins include distribution patterns, environmental factors, and behavioral processes (Mendez et al., 2013). Barriers to individual dispersal and gene flow can exist among different *S*.* chinensis* communities. In the previous studies, significant genetic differentiations of *S*.* chinensis* between the neighboring regions were also detected in Chinese waters (see Zhang et al., 2020; Zhao et al., 2021). Geographic barriers have been found among humpback dolphin populations and were caused by different oceanographic features, such as ocean currents, upwelling, bathymetry, sea surface temperature, primary productivity, and salinity (Amaral et al., 2017; Mendez et al., 2011, 2013). Genetic evidence for Indo‐Pacific marine fauna has shown distinct genetic lineages of several species in the east and the west, including Indo‐Pacific bottlenose dolphins (*Tursiops aduncus*) and humpback dolphins (e.g. Amaral et al., 2017; Keyse et al., 2014; Zhao et al., 2021). Our microsatellite analysis also showed a significant genetic differentiation between WG and AS populations. Historically, the crustal movement of the continental plates and some climatic events may prevent the individual dispersal and gene flow of marine organisms across the Malacca Strait (Zhao et al., 2021). Besides these environmental variables, development of coastal areas may lead to anthropogenic barriers to dispersal and produce the isolated population fragments of inshore dolphins (Brown et al., 2014). It is reported that the coastal development projects have been increasing continuously in the Western Gulf of Thailand (Jutapruet et al., 2015). Therefore, anthropogenic impacts in the coastal habitats of humpback dolphins may be associated with the significant genetic differentiation detected in the Western Pacific and Eastern Indian Ocean sides of the present sampled regions.

Owing to the difficulties associated with biological sample collection, molecular genetics studies on *S*.* chinensis* have often been restricted to using highly degraded DNA samples from museum specimens or dead stranded individuals, which leads to small sample sizes from limited locations. Recently, an increasing number of molecular genetic studies have obtained genetic materials from humpback dolphins and other small coastal dolphins by minimally invasive sampling methods (e.g., Amaral et al., 2017; Brown et al., 2014; Manlik et al., 2019; Raudino et al., 2019; Zhang et al., 2020; Zhao et al., 2021). Future studies may use these methods to increase the sample size and sampling regions, which can better elucidate the genetic patterns and gene flows among different *S*.* chinensis* communities.

## 5 CONCLUSION

In this study, 18 new microsatellite markers with pure and long tandem repeat motifs were isolated from *S*.* chinensis* genomic DNA using Illumina MiSeq sequencing. These polymorphic microsatellites were allocated into 6 multiplex PCR panels and successfully obtained genetic data of 42 *S*.* chinensis* individuals from the Xiamen Bay of China, the Western Gulf of Thailand, and Andaman Sea coast. Our microsatellite evidence, together with mtDNA sequence data reported in the present study area (Zhao et al., 2021), indicate that there are high genetic differentiation among *S*.* chinensis* communities along the Asian coast of the Pacific and the Indian Ocean. The strong genetic structure in *S*.* chinensis* populations may be associated with multiple factors such as geographical distribution patterns, environmental variables, anthropogenic interference, and social behavior. These novel polymorphic microsatellite markers will be useful for future molecular genetics studies on this endangered species and other closely related species.

## CONFLICT OF INTEREST

The authors declare no conflicts of interest.

## AUTHOR CONTRIBUTIONS

**Yufei Dai:** Conceptualization (lead); formal analysis (lead); methodology (lead); software (lead); writing – original draft (lead); writing – review and editing (equal). **Watchara Sakornwimon:** Formal analysis (equal); investigation (equal); methodology (lead); software (lead); writing – original draft (lead). **Rachawadee Chantra:** Investigation (lead); methodology (equal); resources (equal). **Liyuan Zhao:** Formal analysis (equal); software (equal); writing – review and editing (equal). **Fuxing Wu:** Formal analysis (equal); software (equal); writing – review and editing (equal). **Reyilamu Aierken:** Formal analysis (equal); software (equal); writing – review and editing (equal). **Kongkiat Kittiwattanawong:** Conceptualization (lead); project administration (lead); supervision (lead); validation (equal). **Xianyan Wang:** Conceptualization (lead); funding acquisition (lead); project administration (lead); supervision (lead); validation (equal).

## Supporting information

Supplementary Material

## Data Availability

Sequences containing the polymorphic microsatellite loci reported in this paper have been deposited into the GenBank database under the following Accession Numbers: MK766845–MK766870.
