# Supplementary material for: High genetic differentiation of Indo‐Pacific humpback dolphins (Sousa chinensis) along the Asian Coast of the Pacific Ocean
Source: Ecol Evol. 2022 May 7;12(5):e8901. doi: 10.1002/ece3.8901 (PMC9077734; doi:10.1002/ece3.8901)
Supplement: Supplementary file 1 — Supplementary Material [file ECE3-12-e8901-s001.docx]

**Appendix.** Information of 55 *S. chinensis* individuals used in this study.

| **No.** | **Sampling regison** | **Date** | **District** | **Province** | **Latitude** | **Longitude** | **Level of decomposed** |
| --- | --- | --- | --- | --- | --- | --- | --- |
| 1 | WG | 24 Feb 11 | Bang Saphan Noi | PrachuapKiriKhan | 10.988 | 99.496 | Decomposed |
| 2 | WG | 28 May 11 | Muang | Chumphon | 10.360 | 99.209 | Mild decomposed |
| 3 | WG | 19 Nov 11 | Bang Saphan | PrachuapKiriKhan | 11.169 | 99.499 | Fresh |
| 4 | WG | 28 Sep 14 | Donsak | Suratthani | 9.319 | 99.683 | Mild decomposed |
| 5 | WG | 05 Aug 15 | Donsak | Suratthani | 9.324 | 99.683 | Decomposed |
| 6 | WG | 29 Oct 15 | Pranburi | PrachuapKiriKhan | 12.416 | 99.988 | Mild decomposed |
| 7 | WG | 21 Jan 17 | Donsak | Suratthani | 9.339 | 99.661 | Fresh |
| 8 | WG | 14 Feb 17 | Tha Chana | Suratthani | 9.440 | 99.270 | Advanced decomposed |
| 9 | WG | 14 Feb 18 | Thung Ta Ko | Chumphon | 10.098 | 99.149 | Mild decomposed |
| 10 | WG | 14 Apr 18 | Donsak | Suratthani | 9.337 | 99.670 | Advanced decomposed |
| 11 | WG | 29 May 16 | Muang | Rayong | 12.681 | 101.111 | Decomposed |
| 12 | WG | 02 Jun 08 |  | Nakorn Sri Thammarat |  |  |  |
| 13 | WG | 12 Mar 10 | Kanom | Nakorn Sri Thammarat | 9.139 | 99.877 |  |
| 14 | WG | 10 Jun 10 |  | Nakorn Sri Thammarat | 8.798 | 99.943 |  |
| 15 | WG | 27 Aug 10 | Koh Samui | Suratthani |  |  |  |
| 16 | WG | 11 Jun 11 | Muang | Songkhla |  |  | Decomposed |
| 17 | WG | 06 Jul 13 | Panalae | Pattani |  |  |  |
| 18 | WG | 06 Jul 11 | Muang | Songkhla |  |  | Decomposed |
| 19 | WG | 13 May 16 | Kanom | Nakorn Sri Thammarat | 9.183 | 99.875 |  |
| 20 | AS | 02 Oct 10 | Koh Lanta | Krabi | 7.681 | 99.088 | Mild decomposed |
| 21 | AS | 28 Apr 11 | Muang | Satun | 6.597 | 99.919 | Decomposed |
| 22 | AS | 16 Jun 12 | Haad Sumran | Trang | 7.212 | 99.547 | Decomposed |
| 23 | AS | 25 Sep 12 | Haad Sumran | Trang | 7.133 | 99.507 | Mild decomposed |
| 24 | AS | 14 Oct 12 | Haad Sumran | Trang | 7.195 | 99.459 | Decomposed |
| 25 | AS | 05 Nov 12 | Parean | Trang | 6.970 | 99.497 | Fresh |
| 26 | AS | 23 Jan 13 | Sook Sumran | Ranong | 9.368 | 98.394 | Mild decomposed |
| 27 | AS | 26 Feb 13 | Haad Sumran | Trang | 7.188 | 99.554 | Mild decomposed |
| 28 | AS | 26 Feb 13 | Haad Sumran | Trang | 7.038 | 99.489 | Decomposed |
| 29 | AS | 17 Mar 13 | Haad Sumran | Trang | 7.070 | 99.516 | Mild decomposed |
| 30 | AS | 25 Dec 12 | Parean | Trang | 7.125 | 99.587 | Decomposed |
| 31 | AS | 15 Apr 13 | Haad Sumran | Trang | 7.277 | 99.516 | Fresh |
| 32 | AS | 02 Jul 13 | Tha Lang | Phuket | 7.984 | 98.276 | Decomposed |
| 33 | AS | 04 Aug 13 | Haad Sumran | Trang | 7.147 | 99.580 | Fresh |
| 34 | AS | 25 Sep 13 | Parean | Trang | 7.080 | 99.615 | Fresh |
| 35 | AS | 04 Aug 14 | Kuntang | Trang | 7.330 | 99.390 | Decomposed |
| 36 | AS | 20 Aug 14 | Parean | Trang | 7.090 | 99.580 | Fresh |
| 37 | AS | 23 Sep 14 | Kuntang | Trang | 7.242 | 99.417 | Mild decomposed |
| 38 | AS | 01 Nov 14 | Haad Sumran | Trang | 7.066 | 99.452 | Fresh |
| 39 | AS | 10 Mar 15 | Haad Sumran | Trang | 7.112 | 99.578 | Decomposed |
| 40 | AS | 26 May 15 | Haad Sumran | Trang | 7.050 | 99.500 | Decomposed |
| 41 | XM | 04 May 10 | Xiamen | Fujian | 24.472 | 118.045 | Decomposed |
| 42 | XM | 16 Mar 15 | Xiamen | Fujian | 24.624 | 118.440 | Decomposed |
| 43 | XM | 04 Jun 13 | Xiamen | Fujian | 24.552 | 118.386 | Fresh |
| 44 | XM | 25 Mar 16 | Xiamen | Fujian | 24.615 | 118.439 | Mild decomposed |
| 45 | XM | 07 Jun 10 | Xiamen | Fujian | 24.434 | 118.094 | Mild decomposed |
| 46 | XM | 10 Aug 15 | Xiamen | Fujian | 24.427 | 118.146 | Fresh |
| 47 | XM | 15 Sep 13 | Xiamen | Fujian | 24.534 | 118.248 | Mild decomposed |
| 48 | XM | 27 Jun 16 | Xiamen | Fujian |  |  | Decomposed |
| 49 | XM | 03 Aug 13 | Xiamen | Fujian | 24.450 | 118.047 | Decomposed |
| 50 | XM | 13 Aug 15 | Xiamen | Fujian |  |  | Mild decomposed |
| 51 | XM | 24 Feb 16 | Xiamen | Fujian |  |  | Decomposed |
| 52 | XM | 15 Sep 15 | Xiamen | Fujian |  |  | Mild decomposed |
| 53 | XM | 13 May 15 | Xiamen | Fujian |  |  | Decomposed |
| 54 | XM | 18 Jul 12 | Xiamen | Fujian |  |  | Decomposed |
| 55 | XM | 07 Apr 12 | Xiamen | Fujian |  |  | Decomposed |
